# The Familial Aggregation of Cigarette Smoking in Kish, Iran

**Published:** 2012-03-01

**Authors:** A Mansouri, I Alvandi, K Mohammad, H Zeraati, A Fotouhi

**Affiliations:** 1 Department of Epidemiology and Biostatistics, Tehran University of Medical Sciences, Tehran, Iran

**Keywords:** Smoking, Familial aggregation, Pairwise odds ratio, Family-based methods

## Abstract

**Background:**

Based on WHO reports, smoking is an epidemic in developing countries. One of important issues about this behavior is its distribution pattern in family members. The main purpose of this study was to evaluate if cigarette smoking had a tendency to cluster or aggregate in the families and what the determinants were.

**Methods:**

Using a multi-stage random cluster sampling approach, a household survey was conducted in Kish Island in 2009. We used the Alternating Logistic Regressions algorithm to model to show the familial aggregation.

**Results:**

The odds ratio for the aggregation of cigarette smoking between family members was 1.63 (1.29-2.06) which increased to 1.96 (1.50-2.55) after adjustment for demographic factors. There was no significant correlation between siblings' cigarette smoking nor was between spouses but the pairwise odds ratio for parents offspring was significant. In other words, cigarette smoking in at least one of the parents increased the odds of being a smoker in offspring significantly.

**Conclusion:**

The study showed that the smoking behavior aggregated in families significantly. The inter-parent offspring aggregation was the main component of the familial aggregation. Higher education and age-gender interaction were determinants of smoking in the families. The programs for prevention and cessation of this behavior in the community might be more successful if they were designed in a family-based rather than an individual-based approach.

## Introduction

Cigarette smoking remains one of the leading causes of preventable diseases worldwide.[1] It is a known cause of respiratory disorders, cardiovascular diseases, various cancers and many other diseases.[2] The World Health Organization (WHO) has predicted that in the year 2015, smoking will cause 50% more deaths as compared to HIV/AIDS.[3] Based on another report from WHO, 5.4 million people die annually because of cigarette smoking. Smoking is the sixth cause of death in the world; if the trend remains unchanged until 2030, the number of smoking-induced deaths will increase to eight to ten million.[4] Furthermore, tobacco use is an important entry portal for abuse of and dependency on other substances such as alcohol or opioids.[5] Based on WHO reports, smoking is an epidemic in developing countries. In Iran, a survey in the year 2000 showed the prevalence of smoking in males and females to be 26% and 1.4% respectively.[6] In the population of Tehran, it has been estimated that 20.6% of the males and 2.9% of the females were smokers.[4] The Fagerstrom test to estimating the rate of nicotine dependency showed that 55.2% of the Iranian smokers had high dependency, 33.5% had moderate dependency and 11.3% had low dependency.[7]

Thus, the prevalence of smoking in Iran is not trivial or negligible. On the other hand, when a phenomenon is prevalent, it may be seen in more than one member of the family; cigarette smoking is not an exception. For example, Heydari et al. showed that 14.3% of daily smoker students had at least one smoker member in their families.[8] Christophi et al. reported that 67.5% of the smoker adults had smoker parents too.[9] However, the main question will be: "Is the existence of many smokers in a family an accidental finding?" or "Does cigarette smoking has a tendency to cluster or aggregate in families?" We tried to answer these questions in our study. Therefore, the aim of our study was to explore the familial relationships between spouses, siblings and parents offspring regarding the cigarette smoking behavior in Kish, Iran. The existence of more than one smoker member in family is always important for public health programs and plans, regardless of its cause (genetic or environmental).

## Materials and Methods

We used the data from a household survey on irritable bowel syndrome (IBS) which was conducted in Kish Island in 2009. Samples were taken using multi-stage random cluster sampling. Kish has 7 regions. In every region, one alley was randomly selected as a cluster. Then, 50 households in every alley were selected. A total of 2020 people were interviewed. However, because of unreliable responses of children and adolescents regarding their smoking behavior, we performed our analysis only on individuals who were 18 years of age or older (1174 individuals). Interviewers went to the house addresses. They introduced themselves and explained the aims of the study. If the family consented to the interview, questionnaires were completed for every member of family separately; otherwise, a new house address was selected. This approach continued until the sample size was completed.

The outcome variable in our study was cigarette smoking which was inquired through the question:" Do you smoke cigarettes currently? Yes/No". Independent variables were age, gender, marital status, and profession and education level as proxies of the socio economic status.

We employed the SAS V7-9 software for data analysis. We used the ALR (Alternating Logistic Regressions) algorithm to model the correlated data. This approach is very similar to GEE (Generalized Estimating Equations) that is one of the most commonly used modeling techniques for analysis of correlated data. What distinguishes the two approaches is that with the GEE approach, associations between pairs of outcome measures are modeled with correlations whereas with ALR, they are modeled with Odds Ratios (OR).[10] Katz et al. introduce this OR as pairwise Odds Ratio.[11] Pairwise Odds Ratio between the jth and kth responses for the ith subject can be expressed as shown following:

OR_ijk_= P(Y_ij_=1,Y_ik_=1)P(Y_ij_=0,Y_ik_=0) / (Y_ij_=1,Y_ik_=0)P(Y_ij_=0,Y_ik_=1)

This OR displays the odds of being a smoker for kth subject of the family if jth subject of a family is a smoker.

## Results

Demographic characteristics of the participants were shown in Table 1. The mean (standard deviation) age and education years of participants were 38.5 (12.8) and 13.3 (2.7) years, respectively. The prevalence (95% CI) of smoking in this sample was 29.3 (24.5- 25.3), 43.6% (35.6-51.6) in fathers, 14.7% (4.9-24.5) in mothers and 29.6 (22.2-37.0) in their offspring. The distribution of cigarette smoking by demographic variables was shown in Table 1. As shown, cigarette smoking was more common in fathers, in males, in people older than 50 years of age, in office-workers, in divorced people and in low educated people.

**Table 1:** Demographic characteristics and smoking status of participants

****		**All participants**		**Smokers**		
**Variables **		**Number**	**(%)**	**Number**	**(%)**	***P* value**
**Family members**	Father	337	28.8	147	43.6	<0.001
	Mother	341	29.0	50	14.7	
	Children	496	42.2	147	29.6	
**Age groups**	18-25	258	22.0	62	24.0	0.001
	25-50	694	59.1	196	28.2	
	≥50	222	18.9	86	38.7	
**Gender **	Female	532	45.3	131	24.6	<0.001
	Male	634	54.0	211	33.3	
	Unknown	8	0.7	-	-	
**Marital status**	Single	383	32.6	109	28.5	0.322
	Married	742	63.2	216	29.1	
	Divorced	49	4.2	19	38.8	
**Job status**	Home maker	205	17.5	55	26.8	0.648
	Unemployed	185	15.8	52	28.1	
	Office worker	215	18.3	69	32.1	
	Retired	27	2.3	5	18.5	
	Student (school or university)	174	14.8	51	29.3	
	Businessmen	343	29.2	105	30.6	
	Unknown	25	2.1	7	28.0	
**Education years groups**	≥ 5 years	25	2.1	11	44.0	<0.001
	6-12 years	602	51.3	146	24.3	
	≥13 years	547	46.6	187	34.2	

Table 2 displays the distribution of sibling’s smoking by their father's, mother's and parent's smoking status. This table shows that smoking in offspring with the smoker fathers was significantly higher than offspring with non-smoker fathers. Smoking in offspring with smoker mothers was higher than their counterparts with non-smoker mothers. However, this difference was not significant. When smoking was considered in at least one of the parents, cigarette smoking was significantly higher in offspring with at least one smoker parent.

**Table 2:** Smoking prevalence in offspring by parent's smoking status

****		**Offspring Smokers a**		**95% Confidence Interval**		***P* value**
		**Number**	**(%)**	**Lower**	**Upper**	****
**Father smoking**	Yes	74	67.9	57.3	78.5	<0.001
	No	40	27.2	13.4	41.0	
**Mother smoking**	Yes	23	59.0	38.9	79.1	0.056
	No	93	42.5	32.5	52.5	
**Parent smoking b**	Yes	83	64.8	54.5	75.1	<0.001
	No	34	26.0	11.3	40.7	

^a^ smoking in at least one of offspring

^b^ smoking in at least one of parents

The OR for the aggregation of cigarette smoking within family members was 1.63 (1.29-2.06) which increased to 1.96 (1.50-2.55) after adjustment for demographic factors. It means that the existence of cigarette smoking behavior in at least 1 member of the family increases the odds of being a smoker in other members significantly. The adjusted variables in the model were age, gender, marital status, profession, and education level as proxies of socioeconomic status as these variables have been included in the models as main covariates for cigarette smoking in most studies.[12]

We investigated the familial aggregation of cigarette smoking behavior between spouses, between siblings, parents-offspring, mothers-offspring, and fathers-offspring. It was performed in 2 stages: firstly, we estimated all mentioned pairwise ORs without adjustment for demographic variables. Then, we adjusted the effect of age, gender, education, profession and marital status and estimated the pairwise ORs. The results were shown in Table 3. As shown, in both stages, there was no significant correlation between siblings' cigarette smoking nor was between spouses but the pairwise OR for parents-offspring was significant in both stages. In other words, cigarette smoking in at least one of the parents increased the odds of being a smoker in offspring significantly. This relationship not only remained significant after the adjustment, but also became stronger. It should be stated that the pairwise OR could not be applied for father-offspring and mother-offspring due to zero value in one of cells of cross-tabulation.

**Table 3:** Adjusted odds ratio for familial aggregation of smoking by alternating logistic regression model

**Variables **		**OR^a^**	**95% CI^b^**		***P* value**
**Gender**		1.20	0.66	2.17	0.560
**Education**		0.90	0.87	0.95	<0.001
**Age groups:**					
18-25 (reference)		-	-	-	-
25-50		0.97	0.70	1.35	0.830
> 50		0.94	0.56	1.57	0.790
**Age × Gender (interaction)**		0.98	0.95	0.99	<0.001
Odds ratios for familial aggregation of smoking (before and after adjustment)					
	****	**PWOR^c^**	**95% CI**		***P*-value**
**Intra familial **	Before Adjustment	1.63	1.29	2.06	<0.001
	After Adjustment	1.96	1.50	2.55	<0.001
**Inter husbands **	Before Adjustment	0.78	0.45	1.36	0.390
	After Adjustment	1.27	0.74	2.16	0.400
**Inter sibling **	Before Adjustment	1.23	0.81	1.85	0.330
	After Adjustment	1.26	0.80	1.96	0.310
**Inter-parent-offspring **	Before Adjustment	2.19	1.69	2.84	<0.001
	After Adjustment	2.58	1.90	3.49	<0.001

^a^ OR=Odds ratio

^b^ CI=Confidence interval

^c^ PWOR=Pairwise odds ratio

## Discussion

Family studies are one of the most common methods for examination of the family distribution patterns of some of the attributes or disorders.[13] Therefore, we investigated the aggregation of cigarette smoking behavior among first degree relatives in a family-based study. Our purpose was not to estimate the role of genetic and environmental contribution in the development of cigarette smoking behavior in family members but to display the role of family in the aggregation of this behavior.

The studies on twins showed that a variance of 46-72% in starting smoking and 62% in continuing this behavior is due to genetics.[5] On the other hand, Cheng et al. reported that familial aggregation of smoking was the result of genetics, shared environment and interaction between both; they even stated that genetic and environmental factors played partly equal roles in the total variance of cigarette smoking behavior.[14]

In our study, the prevalence of cigarette smoking was 43.6, 14.7 and 29.6 in fathers, mothers and offspring, respectively. The findings, except for the results for mothers, were rather similar to other studies. Miles et al. estimated the prevalence of nicotine dependency as 45.7%, 50%, 20% and 17.8% in fathers, mothers, brothers and sisters respectively[15] while Harakeh et al. reported lower frequencies for family members' cigarette smoking-except for mothers as compared to our study (21.4%, 18.4%, 10.1% and 5.6% in fathers, mothers, older offspring and younger offspring respectively).[16] However, these figures showed that cigarette smoking was not a negligible issue in families and therefore, investigation of its familial patterns can elucidate the role of the family.

Based on Table 3, smoking in offspring with smoker parents was significantly higher than their counterparts with non-smoker parents. Griesbach et al. also showed that cigarette smoking in the children of Finnish, Swedish, Danish, German, Norwegian, Scottish and Australian smoker parents was significantly higher than children of non-smoker parents.[17] As implied, we noted that the intra-familial OR for being a smoker increased from 1.63 (1.29-2.06) to 1.96 (1.50-2.55) after adjustment. Therefore, it is clear that cigarette smoking aggregates in families. In other words, if there is at least one smoker member in the family, the odds for being smoker in other members increases by 1.63 times or 1.96 times (after adjustment). These findings are in line with other studies. For example, Niu et al. showed that the OR for nicotine dependency in first degree relatives of smokers was 2.13 (1.02-4.43).[5] Also, Nurenbrger et al. estimated an OR equal to 2.24 (1.88-2.25) for tobacco dependency in first degree relatives of smokers.[18] These results mean that the family plays an important role in developing or not developing the habit of smoking in its members. We investigated deeper relationships in the family as well. Regarding spouses, we did not find a significant correlation. Familial aggregation OR was 0.78 (0.45-1.36) which increased to 1.25 (0.73-2.15). Although it is difficult to discuss and analyze this strange and insignificant finding, other studies have shown significant relationships between spouses. Cheng et al. found a significant positive correlation between spouses. Based on their study, a smoker spouse has a strong and positive effect on her or his partner.[14] This correlation has been detected for other substances. Regarding cannabis use disorder, Merikangas et al. estimated an OR of 4.4 for spouses.[19] The OR for alcohol use disorders between the spouses was estimated to be 3.8 (1.1-13.02) by another study.[20] To justify these aggregation, Cheng et al. proposed that the spouses had similar attitudes before marriage.[14] Regarding the negative correlation between spouses before adjustment in our study, it can be said that perhaps a smoker person tends to choose his or her spouse from non-smoker individuals.

We did not find a significant relationship between sisters and brothers (siblings) too. OR between them was 1.23 (0.81-1.85) before and 1.26 (0.80-1.96) after adjustment. However, other studies have achieved remarkable results. Bierut et al. estimated the OR for developing the habit of smoking in the sisters and brothers of a smoker individual to be 1.77 (1.48-2.12).20Also, Niu et al. reported an OR of 3.50 (1.65-7.36) for nicotine dependency between siblings.[5]

The most important significant finding in our study was the association between parents and their offspring. The familial OR increased from 2.19 (1.69-2.84) to 2.58 (1.90-3.49) after adjustment, which means that a smoker father or mother has an important influence on his or her children's cigarette smoking behavior. This finding has been approved by several studies. As Mirzazadeh and Haghdoust estimated an OR of 1.72 (1.52-1.94) for the relationship between fathers and their offspring.[21] Christophi et al. concluded smoking in one or both parents increased the odds of developing the habit of smoking in their offspring by 1.52 (1.32-1.74).[9] FitzGerald et al. showed that a smoker parent, specially the father, not only significantly influenced the development of the smoking habit in children but also affected and strengthened the positive association between siblings' smoking.[22] On the other hand, some studies have shown opposite results. For example, Thamson et al. reported that smoking of the parents had a strong negative effect on their children as the relevant OR decreased from 0.60 (0.48-0.71) to 0.39 (0.32-0.40) after controlling for environmental factors.[23] Also, Cheng et al. indicated that mother’s smoking had a strong protective effect on her children. He justified this finding as a result of the cultural and environmental effects of the family.[14]

As mentioned earlier, we noted that familial aggregation of smoking in some levels existed after adjustment for demographic factors. Two reasons can be stated for this observation: first, existence of this aggregation may be due to unknown environmental factors that we did not enter to the ALR models. We considered profession and education as proxies of socioeconomic status but they may be imperfect. If it is correct, we have not controlled the effect of socio economic status completely. Therefore, we suggest more studies in future for closer investigation of this familial aggregation by controlling more environmental factors. Second, existence of this familial aggregation after adjustment for environmental factors, provided that all environmental factors are controlled, may be indicative of the role of genetics. In other words, heredity may be an important factor for developing the habit of smoking in family members.

This study was performed on the cross-sectional data, so we could not speak about the role of parents in their offspring smoking status and longitudinal studies are recommended.

This study showed that the smoking behavior aggregated in families significantly. The inter-parent-offspring aggregation is the main component of the familial aggregation. Higher education and age gender interaction were determinants of smoking in the families. The programs for prevention and cessation of this behavior in the community might be more successful if they are designed in a family-based rather than an individual-based approach.
